# Male long-distance migrant turned sedentary; The West European pond bat (*Myotis dasycneme*) alters their migration and hibernation behaviour

**DOI:** 10.1371/journal.pone.0217810

**Published:** 2019-10-28

**Authors:** Anne-Jifke Haarsma, Peter H. C. Lina, Aldo M. Voûte, Henk Siepel

**Affiliations:** 1 Animal Ecology and Physiology, Institute for Water and Wetland Research, Radboud University Nijmegen, The Netherlands; 2 Naturalis Biodiversity Center, Leiden, The Netherlands; 3 Independent Researcher, Soest, The Netherlands; 4 Wageningen Environmental Science, Animal Ecology Group, Wageningen, The Netherlands; Peking University, CHINA

## Abstract

During autumn in the temperate zone, insectivorous male bats face a profound energetic challenge, as in the same period they have to make energy choices related to hibernation, mating and migration. To investigate these energetic trade-offs, we compared the body mass of male and female pond bats (*Myotis dasycneme*) through the summer season, characterized the known hibernacula in terms of male or female bias, and subsequently compared their population trend during two study periods, between 1930–1980 and 1980–2015. Towards the end of summer, males began losing weight whilst females were simultaneously accumulating fat, suggesting that males were pre-occupied with mating. We also found evidence for a recent adaptation to this energetic trade-off, males have colonised winter roosts in formerly unoccupied areas, which has consequently led to a change in the migration patterns for the male population of this species. As male bats do not assist in raising offspring, males have ample time to restore their energy balance after hibernation. Our results suggest that choosing a hibernacula closer to the summer range not only decreases energy cost needed for migration, it also lengthens the mating season of the individual male. Our findings have important conservation implications, as male and female biased hibernation assemblages may differ critically in terms of microclimate preferences.

## Introduction

In contrast to bird migration, bat migration and its specifics are virtually unknown, such as its relation to summer distribution patterns, or how bats cope with energetic challenges and changing habitat. Bat species in the Northern hemisphere are almost all insectivorous (e.g. [1, 2]). Due to lack of food resources winters are spent in hibernation. In most species, male and female bats live in segregated summer ranges [3–7]. In autumn sexes meet in mating roosts, the male roosts, or the winter roosts [8–10]. The migration behaviour of bats between their summer and winter roosts is unique. Birds and large ungulates move from areas of low or decreasing resources (i.e. food) to areas of high or increasing resources [11, 12]. In contrast, the direction of migration in bats is mostly determined by the location of hibernacula with the suitable physiological requirements [13]. Consequently, bats in the Northern hemisphere are sometimes observed migrating northwards from their summer roosts [14]. Among bats, distances between summer and winter range of more than several hundreds of kilometres are relatively uncommon [15]. Exceptions in Europe are, for example, *Nyctalus noctula* and *Pipistrellus nathusii*, with the longest observed distances of up to 3,000 km [14]. Such distances are still meagre compared to the tens of thousands of kilometres that some bird species of similar size migrate [16]. Nonetheless bat migration has a substantial impact on energy balance [17, 18].

There are profound differences between the energy choices related to hibernacula selection for male and female bats [8]. Migration prior to hibernation not only requires a considerable energy investment, it also involves a long time spent with flying, perhaps alternated with fuel accumulation and time spent waiting for optimal conditions. Mating takes place (almost) exclusively in autumn, female bats store the sperm/ have delayed implantation, and therefore their investment only starts in spring. The migration and mating season overlap, therefore time and energy spent on migration is not available for mating. Although applicable for both males and females, this trade-off is probably more important to males, since lack of in mating activities, combined with mating in suboptimal time and place, may decrease their chances of fathering offspring. As male bats do not assist in raising their offspring, males have abundant time to restore their energy reserves after hibernation. In contrast, female bats begin their gestation after spring emergence (~ March/ April) and are under considerable time pressure to return to their maternity roost and raise their offspring [19]. In order to do this, females have a thriftier hibernation strategy than males, with behavioural changes and microclimate selection focussed on optimizing fat reserves [20, 21].

Males, however, are thought to spend more energy in mating before hibernation, thus creating a trade-off between mating or migrating. Studies on life history trade-offs have tended to focus on female choice, age of first reproduction, in combination with longevity and hibernation choices (e.g. [22, 23]). Several studies have shown sex influenced differences in distribution patterns [5, 24], indicating that reproductive and energetic choices may also play a role in migration behaviour. In this paper, we present an unique example of how choices of the males affect life history.

Our studied species is the pond bat, *Myotis dasycneme*, a middle-sized species, with a specialisation in water trawling [25]. The pond bat is considered a long-distance migrant with a maximum recorded migration of 344 km [14]. Summer roosts of pond bats are almost exclusively found in buildings, while winter roost includes several types of underground sites, such as caves, cellars and mines. In this paper, we characterize how known hibernacula vary between males and females in terms of male or female bias, and subsequently compare their population trend during two study periods, between 1930–1980 and 1980–2015. We predict that energy spent during mating cannot be spent on hibernation. Hence, we identified how the body mass of bats changes during the summer period. We compare migration distance, calculated by using mark recapture data, in the period before and after settlement in the new hibernacula. Based on previous research [26], we hypothesize that the mating, migration and hibernation are trade-offs, and thus we expect that a change in mate site location will lead to a change in migration distance.

## Methods

### Study area, periods and dataset

The study area covered the whole of the Netherlands, Belgium and East Frisia (northwest Germany) (S1 Fig). This area is considered the range of the “West European pond bat population”, an isolated population at the western limit of its distribution range (S2 Fig, based on [27]). Hibernacula of this population are found along the southern and eastern border of its range, in Belgium, North France, West Germany and the southern part of the Netherlands (S3 Fig). Since 1980 hibernacula sites also include several bunkers in Zuid-Holland and Gelderland (the Netherlands). Maternity roosts of this population are predominantly found in the centre of its range, in the Dutch lowlands, with male roost along the edges of the female range [28]. Mating sites are located along high-density migration routes, such as the ‘Afsluitdijk’ (closure dike between Ijsselmeer and Wadden Sea), the coast of Holland and near large rivers.

We defined two study periods, data collected between 1930 and 1980 (‘the historical dataset’) and data between 1980 and 2015 (‘the recent dataset’). The historical data were gathered from literature [27, 29–35] and natural history museum collections. The recent data were gathered from the Dutch Mammal Society and from observations by the first author [28, 36, 37]. These data were supplemented by data gleaned from publications [29, 38–43] and atlases, such as the atlas of the bats of the Netherlands [33], the Flemish region of Belgium [44, 45] and for several atlases of German Federal States [46–48]. Records of marked bats with rings and their biometric data were gathered by the first author and from the former Laboratory for Animal Ecology and Taxonomy of Utrecht University, former office of third author. Results of these former studies were published in several papers, for example [29, 34, 38, 49–51].

### Ringing and biometry

All available mark and recovery data (ringing) of both the historical and recent migration research were digitized. Observations include location and date of capture, species, sex and ring number. The latest observations in the recent dataset also include biometric measurements (forearm length, body mass) and information about age and reproductive status [52]. Body mass was used as an effective proxy of fat mass [53]. We made estimations of the male: female ratio in hibernacula based on recoveries of marked individuals observed in each site.

Bat captures were carried out under license from the Dutch Ministry of Economic affairs, (permits FEF27b/2002/034, FF/75A/2003/150, FF/75a/2006/013, FF/75a/2008/033, FF75a2012/37a and with permissions of all site owners. Ringing was carried out under license of Animal Experiments Committee UDEC 02036, 06058, 07124, 98055 and Alt 09–01). All bats were released within one hour, at the point of capture.

### Winter hibernation surveys

The Netherlands has over 3000 suitable bat hibernacula, almost all manmade (e.g. bunkers, limestone mines, fortresses, castles, ice cellars and basements). Limestone mines have been excavated since the Middle Ages (~ 1300 AD). The first ice cellars were built around 1700 AD [54], fortresses were built during the First World War. The bunkers are the newest sites; built during the Second World War (e.g. [55]). Bats are known to use suitable hibernacula as soon as they are constructed, even while still in use/ during the construction phase.

Hibernation surveys were often included in national monitoring programs [56, 57], in the Netherlands some sites were studied from as early as the beginning of the 20th century. Sites were visited once each winter and systematically searched with torch light until all visible bats were found and identified (without disturbing the bats). Site-specific underrepresentation of the true numbers of hibernating bats is likely, due to variations of internal characteristics of each site (e.g. size, number of cracks and crevices) and the roosting ecology (pond bats are considered to hibernate mostly in cold crevices [58]. Research pioneers, such as Punt and Van Nieuwenhoven [59] and Kugelschafter [60], showed such an error can be considerable.

Despite these potential biases, the data can be used to estimate coarse trends in abundance (e.g. [61–63]). We selected winter roosts with three or more records of three or more pond bats in one or both of the study periods. Only data from sites with long term data series (from the hibernacula in the Dutch provinces of Zuid-Holland, Gelderland and Limburg) were used to analyse trends and annual abundance. Unfortunately, due to a change in regulations (Mining act of the Netherlands), legal access to the Dutch limestone mines needed to carry out monitoring surveys has become problematic since 2011. Hence, we present winter data only up to 2015. Our selection included 59 limestone mines in Limburg and 16 WOII bunkers in Gelderland and 38 in Zuid-Holland (S3 Fig).

We measured the microclimate (wall temperature) in 86 hibernacula frequently used by pond bats. The microclimate was measured at all sites in at least two years. We used a Raytek ST80, a non-contact infrared thermometer (Distance to spot size = 50:1, accuracy = ± 1%, resolution = 0.1°C). In each hibernaculum, observation transects of 50 m length were chosen, which were located randomly throughout each site. All bats present within these transects were matched with the microclimate measured in one point in the centre of this transect.

### Statistical analysis

We calculated annual abundance using General Linear Models (GLMs) with a Poisson error distribution, implemented in the software TRIM [64]. This programme derives the values of missing observations based on previous and succeeding values. We used the linear trend model with all change points included (this is because of the absence of observations in 1949). Non-significant change points were excluded stepwise.

## Results

### Mating effects, weight of males during autumn

**Fig 1:**
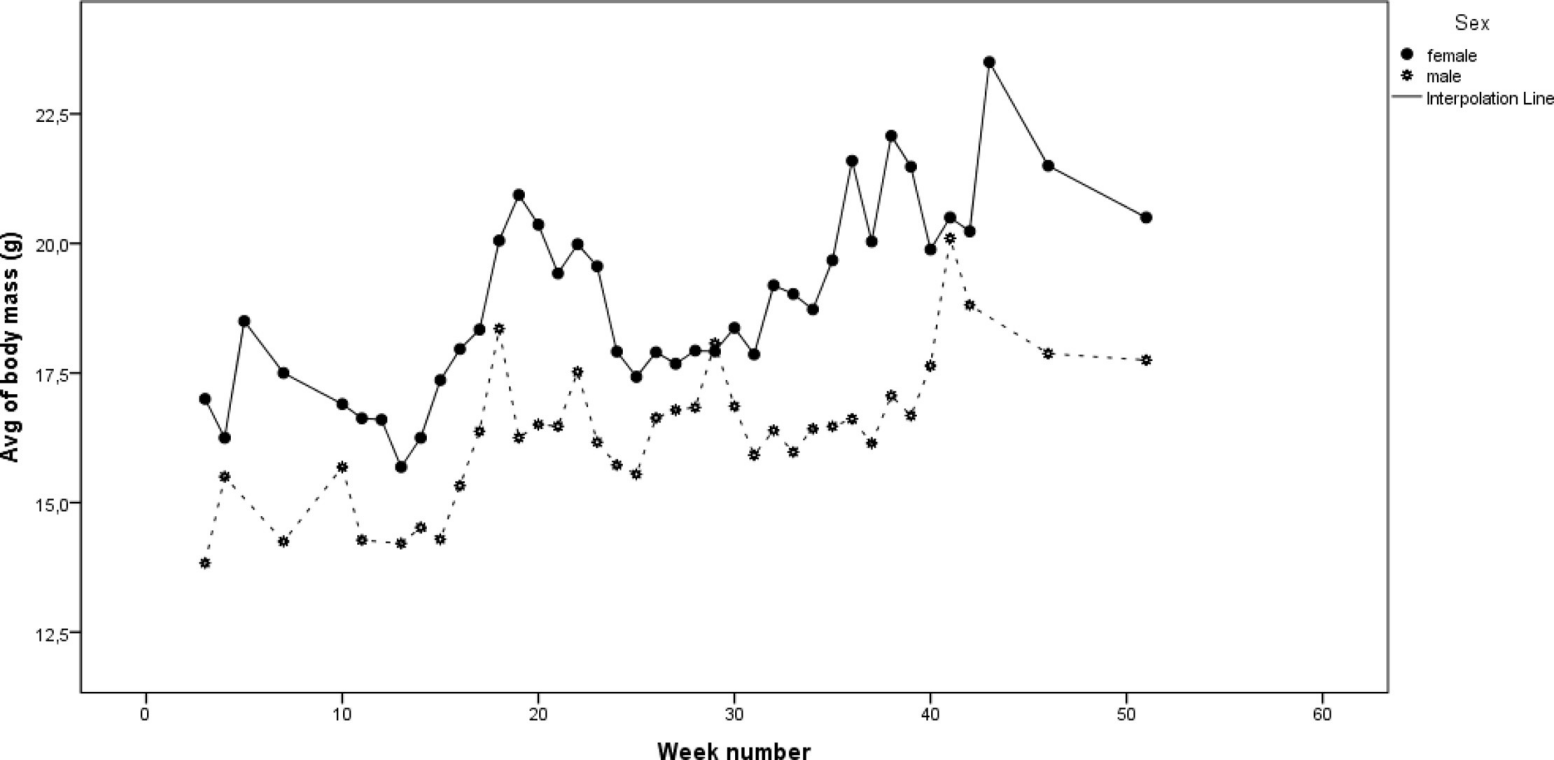
Fluctuations in the body mass of the adult population during the year (data collected in period 2002–2015).

The biometric measurements of adult and/ or sexually mature pond bats showed that male pond bats are on average smaller and lighter than females (body mass (g)/ forearm length (mm) females: 18.9/47.1, males: 16.4/46.4). Analysis of our dataset revealed that changes of fat mass of both sexes do not occur simultaneously, each appears to be related to its respective reproductive cycle (S1 Table, Fig 1). Near week 30 (i.e. end of July), after their offspring is fully weaned, females start accumulating fat reserves. The onset of mating activities starts with the influx of males towards the mating sites (approximately week 28, i.e. mid-July). From this time onwards males become preoccupied with mating activities and as a consequence lose weight. Instead of accumulating fat, the weight of males drops and they start falling behind. Just before the onset of the winter (the week where males begin gaining weight again), approximately 4–8 weeks later than females, males reach their pre-hibernation weight. This difference between male and female fat mass just before hibernation stresses the potential gain males may have in adapting their migrating behaviour.

### Selection of new hibernacula near summer sites

**Fig 2:**
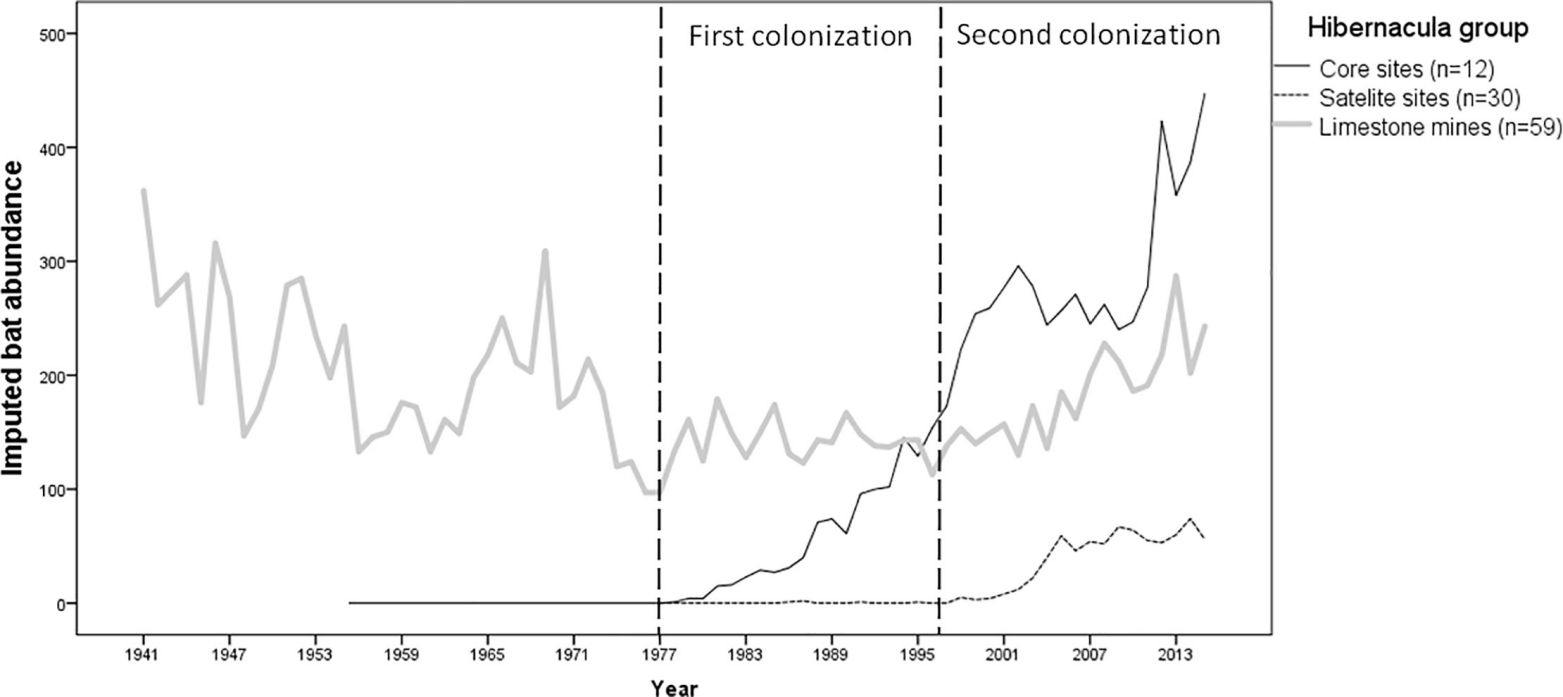
The (imputed) total annual abundance of pond bats in hibernacula in the provinces of Gelderland, Zuid-Holland and Limburg. The three lines represent three groups of hibernacula; core sites (12 roosts, unbroken line), satellite sites (30 roosts, broken line) and limestone mines (59 roost, grey line).

Between 1900 and 1976 pond bats were only found in the limestone mines in Limburg. The first colonization of hibernacula in formerly unoccupied areas occurred in the beginning of the 1980s (Fig 2). Between 1977 and 1986 low numbers of male bats were found in 12 bunkers in the provinces of Zuid-Holland and Gelderland (hereafter referred to as the core bunkers), respectively along the western border and in the centre of the distribution range of the West European population. Although sites adjacent to these core bunkers were visited annually, pond bats remained (nearly) absent until the year 1997 (referred to as the second colonization event). After an exponential increase in both core and adjacent bunkers (14 sites in Gelderland and 28 in Zuid-Holland), today’s numbers are 503 times higher than in the colonization year.

This strong increase is in stark contrast to the population trend observed in Limburg and Southern Belgium. For instance, between 1941 and 1961, the annual counts at 59 sites in Limburg decreased from 350 to 132 individuals. Instead of site abandonment, the average density of animals per site decreased from 6.1 to 3.2 pond bats. In the same period in southern Belgium the hibernation population decreased from an estimated 200 animals to a meagre 20 animals (11.6% decrease based on 58 hibernacula). Since 1980, hibernation populations in Limburg and Southern Belgium remained more or less at the same reduced level after the decline.

### Assemblage in hibernacula seems either male or female biased

We found that the bunkers were predominantly inhabited by males (male:female = 8.2:0.8), while the limestone mines were predominantly female biased (before 1980 male:female = 1.3:3.4, after 1980 approximate male:female = 1.1:5.4).

### Temperature characteristics of winter roosts

**Fig 3:**
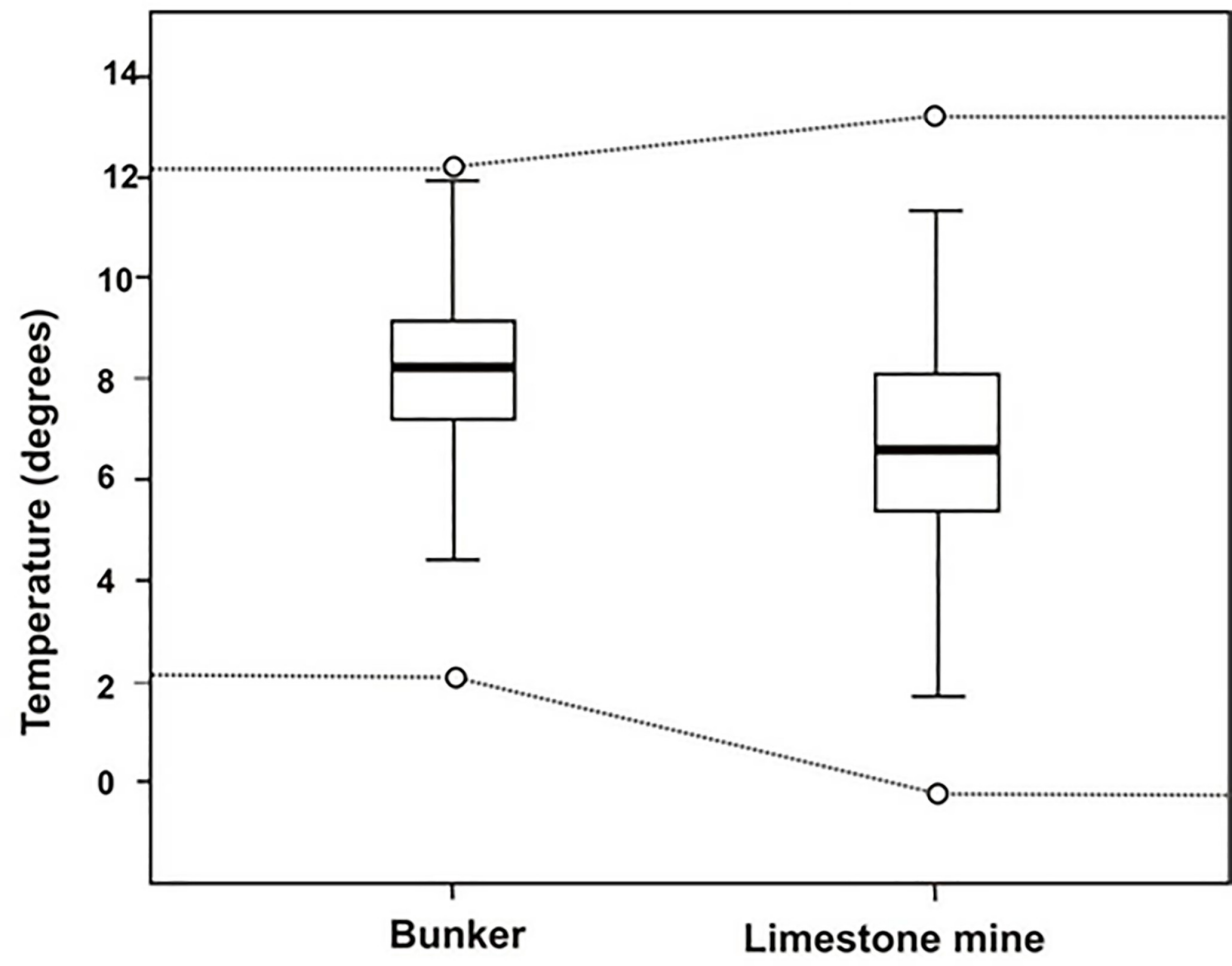
Boxplot showing the microclimate range in the hibernacula and the microclimate near hibernating pond bats in bunkers (54 sites, 1,072 observations near pond bats) and limestone mines (32 sites, 401 observations near pond bats). The box plots show the 25–75 percentiles (box), the 10 and 90 percentile (T). The line within the box indicates the mean. The broken lines indicate the observed temperature range inside both hibernaculum types (1,578 bunker and 1,406 limestone mine temperature observation points).

Bunkers and underground limestone mines have very different types of microclimate. Bunkers are relatively small (surface area 9–3,000 m2, average 196 m2, n = 54) with little ground cover on top of each structure (30 cm– 300 cm). This in contrast to the limestone mines (surface area 135–20,000 m2, average 4387 m2, n = 32) with thick ground cover (100 cm– ≥1,000 cm). The temperature in bunkers is strongly influenced by fluctuations of outside weather conditions, but due to small entrances (¬ minimal air current) and warm winters, very low temperatures were not observed. Entrances of larger mines are much larger and can cause more climate instability, however, in the rear part of such sites the temperature remains static, often 13°C. Pond bats in bunkers seem to hibernate at higher temperature than in limestone mine (appr 8.1 versus 6.0°C, Fig 3).

### Change in migration distance

**Fig 4:**
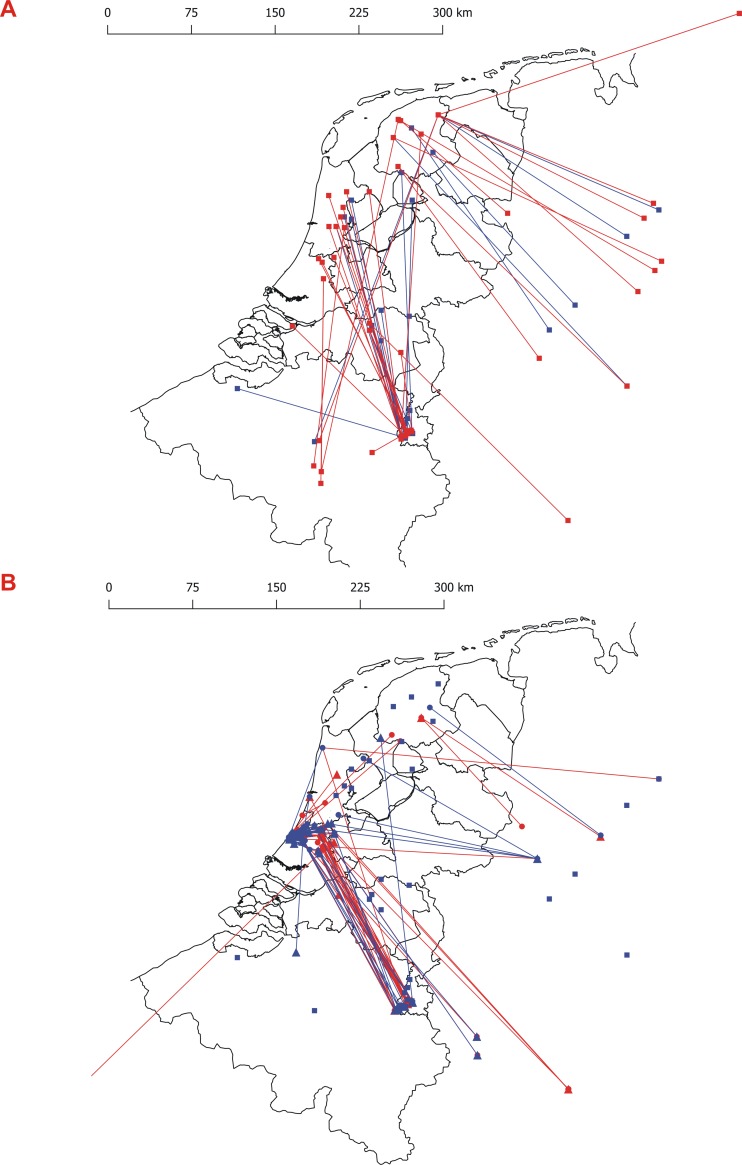
**Map of recoveries from former (a) and recent (b) migration studies.** Both maps are north orientated. In both figures’ males are shown in blue and females in red. The age of an animal is indicated by the shape of the points (square = unknowns age, circles = adult, triangle = sub-adult). The lines connect the point of capture and the point of recovery (adult / unknown age = unbroken line, sub-adult = broken line).

Before 1980, over 3,000 pond bats (>1402 males, >1744 females) were captured and marked in the Netherlands, Belgium and Germany (Table 1). A total of 1,214 pond bats have been ringed in their maternity roosts, marked animals were predominantly females and their offspring. The rest were captured in their hibernacula in Southern Belgium, both Netherlands and Belgian Limburg and the Eifel region. The number of bats captured in the hibernacula was probably underestimated. The reported recoveries of these ringed animals showed that the animals spending the summer in the Netherlands migrated long distances to hibernacula in the Southern Netherlands (Limburg), Belgium (Namur) and in Germany (S2 Table, Fig 4(A)). Animals from the Dutch province of Friesland were often recovered to the south in Southern Limburg in the Netherlands and to the east in Germany with a preference for the Teutoburg forest region.

**Table 1:** Overview of migration behaviour based on historical data (1939–1973) and recent data (2002–2015). The direction of migration is the direction with the summer roosts as starting point (even if bats were originally captured during winter).

Period	Historical data		Recent data	
Sex	Males	Females	Males	Females
Number of marked animals	>1,402	>1744	562	1,165
Average direction (degrees)	128.91	132.07	283.29	199.74
StDev of direction	35.40	35.88	83.64	88.88
Average distance	152.20	191.73	41.05	114.33
StDev of distance	78.17	62.93	91.16	61.25
Maximum distance (km)	296	344	236	309

After 1980 a total of 1,727 pond bats were captured and marked (195 in their winter range and 1,532 in their summer range, Table 1). Of the 193 recaptures, 127 (97 males and 30 females) were found in the bunkers near their summer range (Table 2). Only a small number of animals were recovered at distances greater than 50 km from their original place of capture. These long-distance migrants, both adult and sub-adult, were recovered in hibernacula such as the fortresses in Antwerp, bunkers in Calais (France), several sites in the Teutoburg forest region, limestone mines in Limburg and underground caves in the Eifel (S3 Table, Fig 4(B)). No recoveries were made in the province of Namur (southern Belgium). Unfortunately, the absolute number of recent long-distance recaptures in Limburg is probably underestimated, due to restricted legal access to the limestone mines since 2011.

**Table 2:** Details of migration behaviour based on historical data (1939–1973) and recent data (2002–2015). The numbers in parentheses are the recovered fraction of the marked population, calculated for each gender. Short distances are distances less than 50 km between the summer and winter roost, long distances are more than 50 km.

Period	Historical data		Recent data	
Sex	Males	Females	Males	Females
N of long distance migrants (age unknown)	24 (< 1.7%)	31 (<1.7%)		
N of long distance migrants (adults)			6 (1.1%)	29 (2.4%)
N of long distance migrants (sub-adults)			15 (2.6%)	16 (1.4%)
**Total long distance**	**24**	**31**	**21**	**45**
N of short distance migrants (age unknown)	0 (0%)	0 (0%)		
N of short distance migrants (adults)			60 (10.7%)	19 (1.6%)
N of short distance migrants (sub-adults)			37 (6.5%)	11 (0.9%)
**Total short distance**	**0**	**0**	**97**	**30**

## Discussion

Our study indicates that there is an evidence for the energetic trade-off between mating, migration and hibernation. We found several likely confounding factors indicating this trade-off, such as different pre-hibernation biometric measurements, the colonization of new hibernacula and subsequent change in migration distance, and the different hibernation temperature observed between bunkers and limestone mines. The overall ratio of males and females present in the hibernacula investigated in this study was not uniform, we found typical male and female assemblages. The possible preference of females for limestone mines, supports the thrifty female hypothesis [20, 65] and could explain why females continue to hibernate in limestone mines, while males changed their hibernation sites. The typical migration distance in the first study period (1930–1980) far exceeds that of the distance during the second study period (1980–2015). Therefore, in accordance with the results of this study, we argue that males after spending energy on mating, prefer to hibernate locally. Indeed, we find a strong increase in the population size in the local hibernacula. Taken together, these results indicate that a trade-off between hibernation, mating and migration is present.

Female biased migration is more common among temperate zone bat species, where females migrate to natal maternity roosts and males remain near over-wintering sites [24, 66, 67]. Several other authors have shown that energy spent during migration has a large impact on their energy balance [68, 69] and thus their life history. Bats seems very adept in optimizing their migration strategy, spending as little time as necessary in migration [17]. The results presented in this paper perhaps can lead to new insights how migration, mating and hibernation interact. Our long-term dataset illustrating settlement of new (before 1945 not existing) local hibernacula, and a change of migration patterns, is unique. We assume sedentary males benefit in terms of energy and time, indicating this change in migration is perhaps led by male choices. Our study exemplifies how bats were able to adapt, within a 40-year timeframe, to a new habitat. Unfavourable anthropogenic activities in foraging habitats and along migration routes are happening with a rapid tempo. Our results may help define conservation priorities and timelines, to prevent endangered bats from going extinct.

Pond bats seem promiscuous, as many individuals change mating groups over years (own data). Females tend to visit the males in their mating sites. In some species multiple maternity colonies make use of one mating site, both throughout the mating season and on individual nights [70]. In other species, such as *Myotis nattereri*, it has been shown that females from a single maternity colony attend multiple mating sites [71]. Other studies show that females visit these mating sites only for one or several nights, then return to their maternity roost [72, 73]. During a telemetry project on pond bats, not linked to this study, the first author observed a similar pattern of alternating use of maternity and mating sites (unpublished data). This mating system is only possible when mating sites are within a short flying distance of maternity sites. A similar relationship between mating and maternity sites is also found with other migratory bat species [74]. While pond bats are considered long distance migrators, with males settling in local hibernacula, we assume they managed to reinstall this mating system.

The trade-off between mating and migration is also seen in other mammalian clades, such as red deer and seals (e.g. [75, 76]). In such systems males spend a lot of energy defending females and are considered territorial. Most male bats do not defend their females, but they can be territorial. Several bat species perform a lek-like mating a behaviour, known as swarming [77, 78, 79]. Swarming behaviour, with intense flight activity, circling in and around the entrance of a site, is considered an interspecies social event and besides mating is also believed to play an important role in the assessment of hibernaculum suitability [80] and/ or information transfer regarding its location [81, 82]. Unlike other bat species, pond bats tend to spend little time swarming in front of the entrance, and instead spend more time outside making long patrol flights to and from the entrance (with flight paths of > 500 m length, personal observations). The presence of large groups of pond bats in the bunkers in Zuid-Holland in the period August–October suggests that they spend their time in temporary harems, like *Myotis myotis* [83]. Given that we do not have individualized data of the behaviour of male bats, we cannot determine the exact nature of how they spend their energy. Future studies into the mating behaviour of the pond bat can help draw this conclusion.

Based on the biometric data, it can be understood that males have the largest gain in the change of hibernacula closer to the summer range, as their fatting up period between mating season and hibernation is shortest. On population level, their trade-off between the risk of choosing a new, maybe less suitable hibernaculum, and a longer trip to the well-known hibernacula seems to pay off, as their numbers are increasing rapidly. Possible additional advantages are the increase in the length of the mating season (and thus greater chance of siring offspring) and/ or a strong first male advantage. Other studies suggest the number of offspring sired is significantly affected by the position in the mating sequence [84], so this increased first male advantage may be a significant gain. The first male advantage is also known with another bat species, such as *Rhinolophus ferrumequinum* [85].

## Conservation implications and applications

Our findings have several notable implications for monitoring surveys. The non-uniform distribution of males and females across hibernacula can influence the monitoring results and lead to false conclusions. Whereas a change in population size in a female site may reflect the trend during the summer, similar changes in population size in a male biased site may reflect something else entirely. Considering the high site fidelity observed in European bat species (e.g. [82, 86]), exchange between two or more hibernacula seems unlikely. We assume a new generation of males is choosing to hibernate locally and that the differential survival of the two ‘male’ strategies is slowly resulting in a change in population estimates in both regions. Therefore, we advise to form conclusions about observed changes in population trend based on the results presented in this paper.

The energetic arguments presented in this paper confirm that the added function of hibernacula for a population of bats as a whole can differ; from male biased assemblages with a predominant role in mating to females biased assemblages with a predominant role for stable hibernation. The energetic advantages can also lead to a change in females’ behaviour, causing a new change in functions. In accordance to their function, such sites may also differ critically in terms of their conservation value for the corresponding species. In addition, protection measurements and microclimate preferences for such sites may also differ accordingly.

## Supporting information

S1 Fig The range of the West European pond bat population. The shaded areas indicate the areas where the bulk of the surveys were carried out.
(TIF)

S2 Fig The distribution of the pond bat in Europe (country boundaries are only indicative). Within the whole range of the species distribution seven groups can be separated.
A The Netherlands, Belgium and Northwest Germany (~the West European population),
B Jutland Peninsula,
C Central European lakelands,
D The Baltic States,
E Ural Mountains (hibernacula),
F Volga Valley (summer nurseries),
G Hungary and Romania.
(JPG)

S3 Fig The distribution of hibernacula used by the western pond bat population. These are sites with three or more records of pond bats in one or both study periods. We identified four roost categories: Roosts which have been used ever since 1900 (= green squares), roosts used only between 1900–1980 (= open black squares), roosts occupied after 1980 (= purple circles), roosts occupied after 1997 (= blue asterisks). Detailed maps, all with the same enlargement, of the clusters in the provinces of Zuid-Holland (1), Gelderland (1) and Limburg (3) are provided.
(TIF)

S1 Table Summary of the average weight of pond bats over the study period. The weight is averaged per week. The table gives average weight of females, males both adults and juveniles.
(XLS)

S2 Table Mark and recapture data from the historical dataset. (XLS)

S3 Table Mark and recapture data from the recent dataset. (XLSX)
