# Supplementary figures and images for: Male long-distance migrant turned sedentary; The West European pond bat (Myotis dasycneme) alters their migration and hibernation behaviour
Source: PLoS One. 2019 Oct 28;14(10):e0217810. doi: 10.1371/journal.pone.0217810 (PMC6816563; doi:10.1371/journal.pone.0217810)

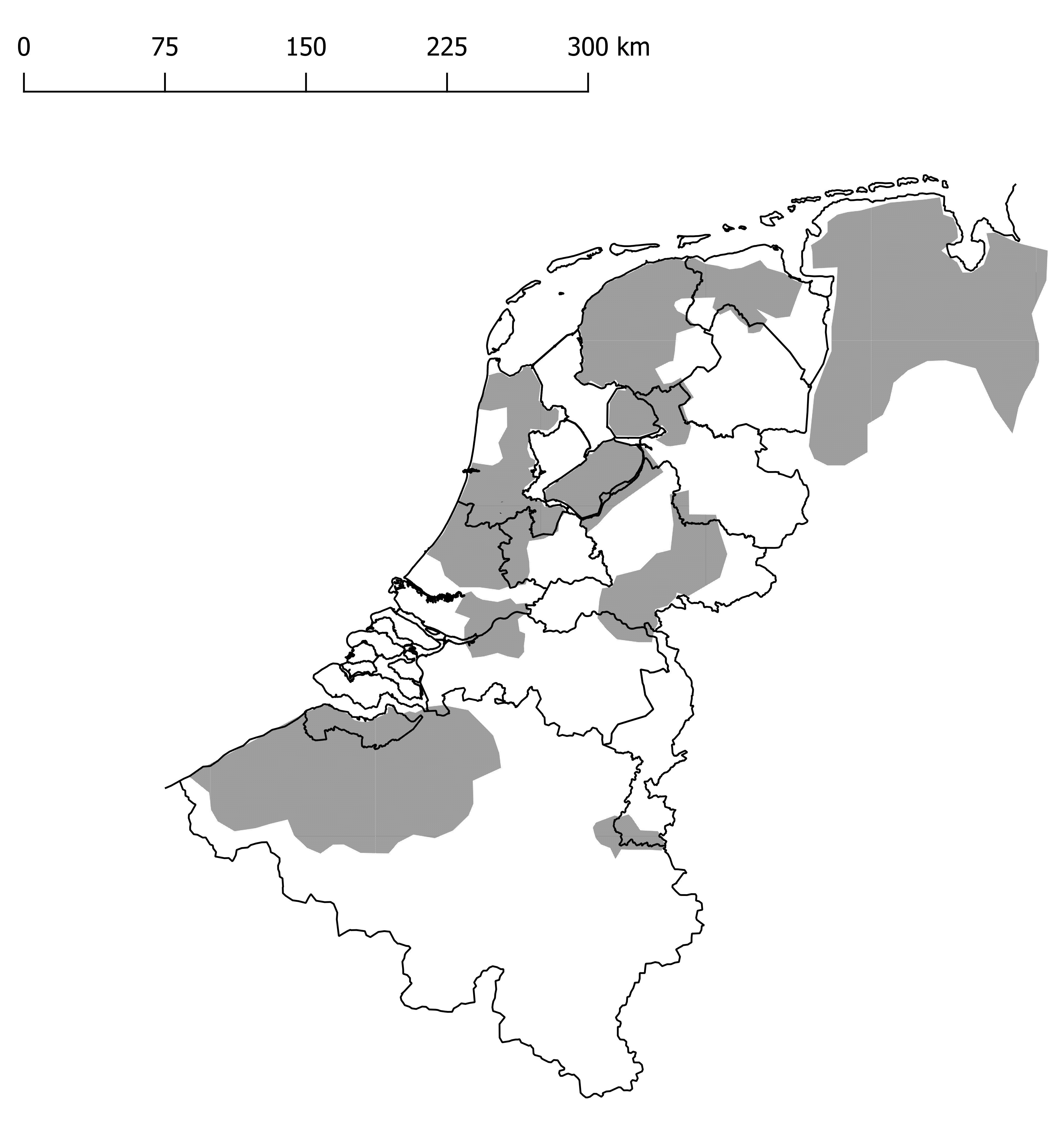

Supplement: S1 Fig — The shaded areas indicate the areas where the bulk of the surveys were carried out. (TIF) [file pone.0217810.s001.tif]

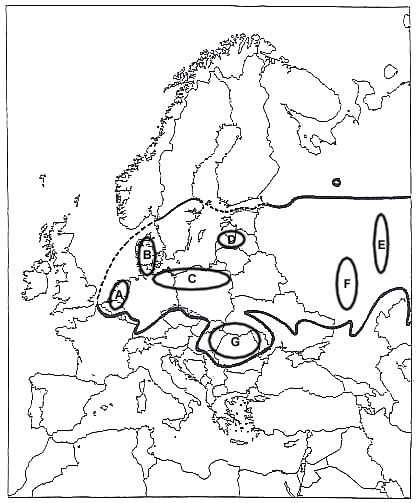

Supplement: S2 Fig — Within the whole range of the species distribution seven groups can be separated. A The Netherlands, Belgium and Northwest Germany (~the West European population), B Jutland Peninsula, C Central European lakelands, D The Baltic States, E Ural Mountains (hibernacula), F Volga Valley (summer nurseries), G Hungary and Romania. (JPG) [file pone.0217810.s002.jpg]

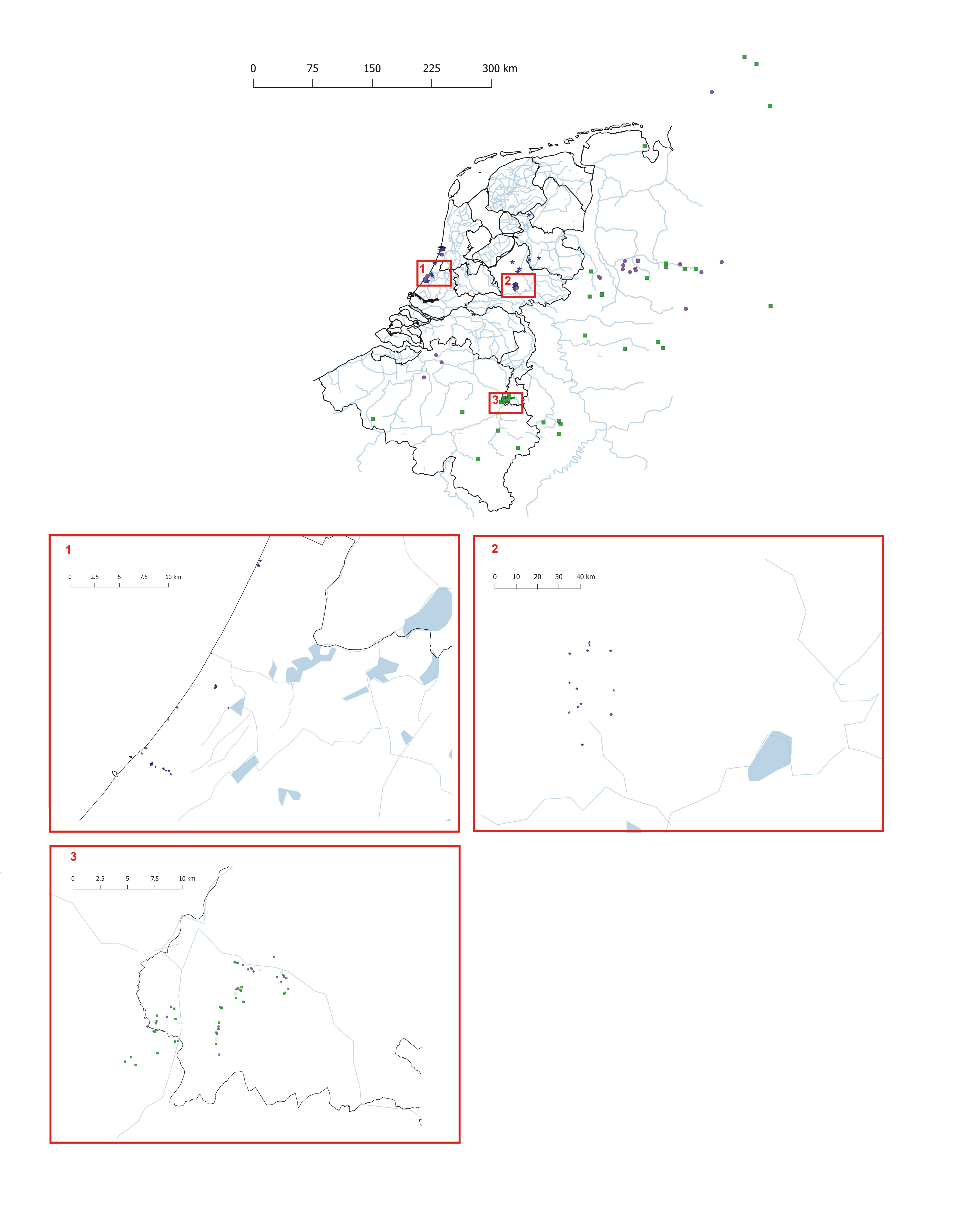

Supplement: S3 Fig — These are sites with three or more records of pond bats in one or both study periods. We identified four roost categories: Roosts which have been used ever since 1900 (= green squares), roosts used only between 1900–1980 (= open black squares), roosts occupied after 1980 (= purple circles), roosts occupied after 1997 (= blue asterisks). Detailed maps, all with the same enlargement, of the clusters in the provinces of Zuid-Holland (1), Gelderland (1) and Limburg (3) are provided. (TIF) [file pone.0217810.s003.tif]
